# Regenerant *Arabidopsis* Lineages Display a Distinct Genome-Wide Spectrum of Mutations Conferring Variant Phenotypes

**DOI:** 10.1016/j.cub.2011.07.002

**Published:** 2011-08-23

**Authors:** Caifu Jiang, Aziz Mithani, Xiangchao Gan, Eric J. Belfield, John P. Klingler, Jian-Kang Zhu, Jiannis Ragoussis, Richard Mott, Nicholas P. Harberd

**Affiliations:** 1 Department of Plant Sciences, University of Oxford, South Parks Road, Oxford OX1 3RB, UK; 2 Plant Stress Genomics Research Center, King Abdullah University of Science and Technology, Thuwal, Saudi Arabia; 3 The Wellcome Trust Centre for Human Genetics, University of Oxford, Roosevelt Drive, Oxford OX3 7BN, UK

## Abstract

Multicellular organisms can be regenerated from totipotent differentiated somatic cell or nuclear founders [1–3]. Organisms regenerated from clonally related isogenic founders might a priori have been expected to be phenotypically invariant. However, clonal regenerant animals display variant phenotypes caused by defective epigenetic reprogramming of gene expression [2], and clonal regenerant plants exhibit poorly understood heritable phenotypic (“somaclonal”) variation [4–7]. Here we show that somaclonal variation in regenerant *Arabidopsis* lineages is associated with genome-wide elevation in DNA sequence mutation rate. We also show that regenerant mutations comprise a distinctive molecular spectrum of base substitutions, insertions, and deletions that probably results from decreased DNA repair fidelity. Finally, we show that while regenerant base substitutions are a likely major genetic cause of the somaclonal variation of regenerant *Arabidopsis* lineages, transposon movement is unlikely to contribute substantially to that variation. We conclude that the phenotypic variation of regenerant plants, unlike that of regenerant animals, is substantially due to DNA sequence mutation.

## Highlights

► Regenerant *Arabidopsis* lineages display heritable phenotypic variation ► Regenerant *Arabidopsis* lineages display elevated genome-wide DNA sequence mutation ► Regenerant DNA sequence mutations comprise a distinct molecular spectrum ► Regenerant base substitution mutations confer heritable phenotypic variation

## Results

### Regenerant *Arabidopsis* Lineages Display Somaclonal Variation

*Arabidopsis* is a genetic model plant with a condensed genome (∼120 Mb). We reasoned that analysis of regenerant *Arabidopsis* plant genomes at single-base resolution might reveal genetic changes conferring somaclonal variation. In vitro regeneration of *Arabidopsis* is achieved via two-stage culture of root explants [8]. First, a pluripotent cell mass (callus) forms via activation of a lateral root genetic developmental program in pericycle cells of explants grown on auxin-rich medium [9, 10]. Subsequently, shoots or roots develop from callus grown on media containing specific auxin:cytokinin concentration ratios. The de novo induction of shoot meristems from small clusters of progenitor callus cells [11] leads eventually to the regeneration of an entire plant.

**Figure 1:**
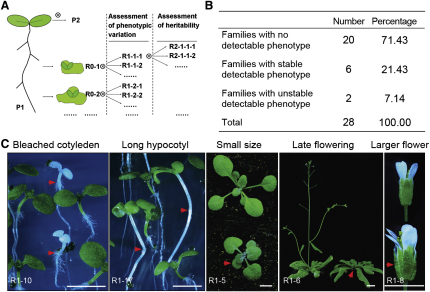
Regeneration-Induced “Somaclonal” Variation in *Arabidopsis* (A) A root explant from a single parental (P1) plant was the source (see [8]) of all R0 regenerant and subsequent generation plants (also see Table S1). (B) Frequency (number and percentage) of R1 families segregating phenotypic variants (also see Figure S1). (C) Selected phenotypic variant R1 plants (segregating variant plants or organs highlighted with red arrows). Scale bars represent 0.5 cm.

We first determined whether regenerant *Arabidopsis* lineages display heritable phenotypic somaclonal variation comparable with that seen in other species. Twenty-eight (R0) plants were regenerated from explants from a single *Arabidopsis* (Columbia laboratory strain; Col-0) root [8] (Figure 1A), and phenotypic variation was assessed in resultant self-pollination generated (R1) families (Figures 1A–1C; see also Figure S1 available online). Variant phenotypes were detected in 8 of the 28 lineages (Figures 1B and 1C; see also Table S1). Two of these phenotypes were not stably heritable (Figure 1B; see also Figure S1 and Table S1) and probably were conferred by unstable epigenetic change. In six further cases, phenotypes were stably heritable and segregated within R1 families, with segregation ratios approximating to Mendelian expectations for single-gene recessive mutations that had been heterozygous in the preceding R0 plant (Figure 1B; see also Table S1; data not shown). Thus in vitro regeneration of *Arabidopsis* plants results in a high frequency of heritable phenotypic variation and provides a general model for the study of plant somaclonal variation.

### Regenerant *Arabidopsis* Lineages Display a Characteristic Spectrum of Genome-wide DNA Sequence Mutation

**Figure 2:**
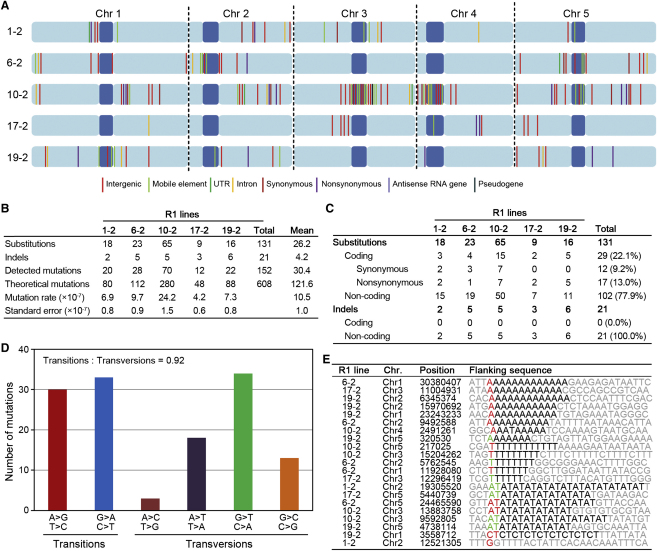
Genome-Wide Analysis of Mutations in Regenerant *Arabidopsis* Lineages (A) Diagrammatic representation of the distribution of mutations on the chromosomes of five independent R1 plants. Centromeres are represented in dark blue [13]. (B) Frequency of base substitutions and indels in sequenced R1 plants, with individual mutation rates. “Detected” and “Theoretical” are defined in the text. (C) Frequency of genomic location subcategories of base substitution and indel mutations. (D) Distribution of specific classes of regenerant base substitution mutation. (E) Regenerant indel mutations: locations and flanking sequences. Homopolymeric or polydinucleotide stretches are in bold. Colors highlight deleted (red) or inserted (green) bases. See Figure S2 and Table S2.

We reasoned that the somaclonal variation exhibited by regenerant *Arabidopsis* lineages might be associated with an increase in genomic DNA sequence mutation. We therefore next determined the genome-wide extent and molecular spectrum of DNA sequence mutations in regenerant *Arabidopsis* lineages, using 76 base pair paired-end Illumina “next-generation” DNA sequencing (Figures 2A–2E). DNA samples from the progenitor P1 plant (Figure 1A; progenitor genome) and from five individual R1 plants (Figure 1A; regenerant genomes) were sequenced to a coverage depth of between ∼22× and ∼30× per sample (Figure S2A). For each sample, high-quality uniquely mapped reads covered ∼116 million of the ∼120 million base pairs of the Col-0 TAIR9 reference genome (see Experimental Procedures), and most uncovered regions were located either in centromeres or telomeres (Figures S2A and S2B; Experimental Procedures). We detected 152 novel regenerant single-base substitution (SBSs) and short insertion or deletion (indel) mutations in R1 plants (mutations not present in the P1 progenitor) using a scheme summarized in Figure S2C. In addition, because previous work showed that somaclonal variation can be associated with larger-scale indels and chromosomal abnormalities (e.g., [12]), we exploited the “paired-end” property of Illumina Genome Analyzer data to detect such larger-scale events in R1 plants (see Experimental Procedures; Figures S2E–S2K). Despite exhaustive searches, no large indels or gross chromosomal abnormalities were detected in any of the five R1 plants representing regenerant *Arabidopsis* lineages.

The 152 regenerant DNA sequence mutations comprised 131 SBSs and 21 small indels (≤2 bp) (“Detected mutations” in Figures 2A and 2B; Table S2A). Sample sets of detected SBSs and indels were confirmed by capillary sequencing (see Experimental Procedures). Regenerant mutations were apparently evenly spread between chromosomes (Figure 2A; see also Figure S2L). These de novo mutations would have been heterozygous when they first arose and would hence have had a 25% chance of being homozygous in individual R1 plants of the subsequent (self-pollination generated) generation. Because we were detecting homozygous mutations only (see Experimental Procedures and Figure S2C), and because we expected that only ∼25% of regenerant mutations would have become homozygous in the R1 generation, we accordingly estimated the actual number of regenerant mutations by multiplying the detected mutations by four (“Theoretical mutations” in Figure 2B). We hence derive somaclonal mutation frequencies of between 4.2 × 10^−7^ and 24.2 × 10^−7^ mutations per site in the five regenerant lineages (Figure 2B; see Experimental Procedures). The mutation rate thus increased between 60× and 350× in the regenerant lineages versus the background “spontaneous” mutation rate observed in sexually propagated *Arabidopsis* (∼7 × 10^−9^ mutations per site per generation [13]).

SBSs are the most common category of regenerant mutation (Figures 2B and 2C). However, the ratio of regenerant transitions:transversions (63:68 = 0.92) is very different to that (2.41) seen in sexually propagated plants [13], as are the relative frequencies (molecular spectrum) of individual base substitution classes (Figure 2D; see also Figures S2M–S2P). In addition, regenerant plants carried an elevated frequency of indel mutations (Figure 2E). Intriguingly, nearly all regenerant indels occurred in homopolymeric or polydinucleotide stretches, which is not the case for small indels arising spontaneously in sexually propagated plants [13] (Figure 2E; Figures S2Q and S2R; see also Table S2B). Short read “next-generation” sequencing is relatively poor at reporting indels in simple sequence repeat regions (for further discussion see [13]), which might explain why the small indel mutation rates we determined are less than those previously determined by a different method for sexually propagated plants [14]. Nevertheless, our results show an increased frequency of small indels in regenerant plants at those sites we were able to assay (Table S2B; Figures S2Q and S2R). In summary, the overall distinctiveness of the regenerant mutational molecular spectrum (SBSs and indels) implies that the elevation in mutation frequency observed in regenerant plants cannot simply be attributed to an accelerated accumulation of the same kinds of mutations as arise spontaneously in sexually propagated plants.

### Regenerant Base Substitution Mutations Confer Somaclonal Variant Phenotypes

**Figure 3:**
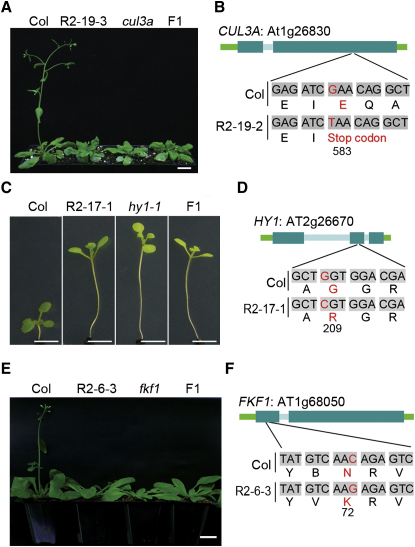
Nonsynonymous Base Substitutions Confer Somaclonal Variant Phenotypes (A) 7-week-old plants, genotypes as indicated. F1 progeny of a cross between a *cul3a* reference allele and R2-19-3 were late flowering, indicating that R2-19-3 was homozygous for a novel mutant *CUL3A* allele. (B) Phenotype-causal base substitution mutation in *CUL3A* (to stop codon; homozygous in R2-19-2). (C) 10-day-old plants, genotypes as indicated. F1 progeny of a cross between the *hy1-1* reference allele and R2-17-1 exhibited a long hypocotyl, indicating that R2-17-1 was homozygous for a novel mutant *HY1* allele. (D) Phenotype-causal base and amino acid substitution in the R2-17-1 *HY1* allele. (E) 45-day-old plants, genotypes as indicated. F1 progeny of a cross between the *fkf1* reference allele and R2-6-3 were late flowering, indicating that R2-6-3 was homozygous for a novel mutant *FKF1* allele. (F) Phenotype-causal base and amino acid substitution in the R2-6-3 *FKF1* allele. Numbers in (B), (D), and (F) represent amino acid position in protein sequence affected by mutations. Scale bars represent 1.0 cm in (A) and (E) and 0.5 cm in (C). See Figure S3.

Of 29 regenerant SBSs affecting protein-coding sequence, 17 were nonsynonymous mutations that alter the amino acid sequence of proteins (Figure 2C; Table S2A). In contrast, none of the indels affected protein-coding sequence (Figure 2C), suggesting that SBSs may be a major genetic cause of somaclonal variation. Indeed, one of the five genome-wide sequenced R1 plants, R1-19-2, carries a protein-truncating SBS in *Cullin 3A* (a locus at which mutant alleles confer late flowering [15]), and a late-flowering phenotype was indeed conferred by this SBS (Figures 3A and 3B). Further genetic complementation and molecular analysis of additional regenerant lines displaying long hypocotyl (R2-17-1; Figures 3C and 3D; Figure S3A) and late flowering (R2-6-3; Figures 3E and 3F; Figure S3B) phenotypes established that they respectively carried novel mutant *HY1* (mutant alleles confer an elongated hypocotyl [16]) and *FKF1* (mutant alleles confer late flowering [17]) alleles. Capillary sequencing of these mutant alleles identified nonsynonymous SBS mutations in the protein-encoding sequences of *HY1* (Figure 3D) and *FKF1* (Figure 3F). While a previous report identifies base substitution mutation as a source of regeneration-associated phenotypic change [18], our findings demonstrate that such mutations are actually a major contributor to somaclonal variation.

### Transposed Mobile Genetic Elements Not Detected in Sampled Regenerant *Arabidopsis* Lineages

**Figure 4:**
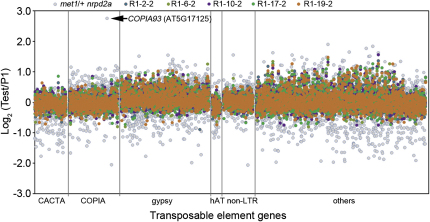
Transposed Mobile Elements Are Not Detected in Regenerant Plants Dot scatter-plot showing log_2_ coverage ratio of reads from R1 lines and from *met1*/+ *nrpd2a* ([20]; colors as indicated) versus P1 reads. Dots cluster around 0, orange predominates because R1-19-2 data were entered last. Each dot represents one of 3,321 transposable elements (grouped in CACTA, COPIA, etc., families). The arrowed dot represents transposition of *AtCOPIA93* [20]. Duplicative transposition would be expected to result in a log_2_ score value of 1. IGV scanning of all dots with value >0.75 (see Experimental Procedures) indicated the absence of detectable transposition during plant regeneration. See Figure S4 and Table S3.

The *Arabidopsis* genome contains multiple mobile genetic elements (transposons), whose activity is normally held in check by DNA methylation and associated epigenetic regulation [19, 20]. Because increased transposon activity has been linked to DNA sequence mutations in rice (*Oryza sativa*) tissue culture [21], we next sought to detect transposed transposons in regenerant *Arabidopsis* lineages, with depth of read coverage as a measure of the increased copy number caused by transposon amplification (see Experimental Procedures). Comparative analysis of the read coverage representation of CACTA, COPIA, gypsy, hAT, non-LTR, and other transposon classes (representative of 3,321 *A. thaliana* transposons, retrotransposons, and other putative mobile elements; Table S3) in whole-genome sequence data from the five R1 regenerant plants and from an epigenetically compromised positive control *met1/+ nrpd2* mutant line (versus P1 data) revealed only the previously described novel *AtCOPIA93* retrotransposition in *met1/+ nrpd2* [20] and no detectable novel transposon amplification in any of the regenerant lines (Figure 4; Figure S4). A second strategy for detection of novel transposon insertions in regenerant lines also detected nothing (see Experimental Procedures). We conclude that insertion of transposons into genes is unlikely to contribute significantly to the genetic variation underlying somaclonal phenotypic variation in regenerent *Arabidopsis* plants.

## Discussion

Here we have shown that regenerant *Arabidopsis* plant lineages display extensive phenotypic somaclonal variation (Figures 1A–1C) and an elevated frequency and characteristic distribution of DNA sequence molecular mutation classes (Figures 2A–2E). We have also shown that the increased base substitution frequency in regenerant plants substantially explains somaclonal phenotypic variance (Figures 3A–3F). Although previous reports have described relatively high frequencies of gross chromosomal abnormality in somaclonal variant lines [12] and transposon movement during in vitro cell culture and/or in regenerant plants (e.g., [21, 22]), we detected neither in our regenerant *Arabidopsis* lineages (Figure 4). It is possible that species differences in genome architecture or the relatively short callus phase duration (∼1 week) in our experiments can explain this apparent discrepancy. We found additional phenotypic variation in regenerant *Arabidopsis* lineages that is probably attributable to variable outcomes of epigenetic reprogramming (Figure 1B; Figure S1), indicating that both genetic and epigenetic phenomena contribute to plant somaclonal variation.

Although clonal regeneration of both plants and animals causes phenotypic variability, our observations suggest that the underlying causes of this variability may differ between the two kingdoms. While we have shown that plant somaclonal variation is substantially due to an increased rate of DNA sequence mutation, regeneration of animals via somatic cell nuclear transfer does not detectably increase mutation rate [23]. Thus, although DNA sequence change explains much of plant somaclonal variation, variability in epigenetic reprogramming of gene expression substantially explains the phenotypic variability of clonally regenerant animals [2, 24].

We here show that the molecular spectrum of regenerant plant mutations distinguishes them from those arising spontaneously in sexually propagated plants [13]. There are two possible nonexclusive explanations for this observation. First, callus phase growth and/or in vitro regeneration from tissue culture might be inherently mutagenic. This first explanation may be supported by previous observations that somaclonal variant phenotype frequencies increase in proportion to the duration during which cells are maintained in tissue culture [25]. Second, mutations in regenerant plants might reflect somatic mutations that existed in the cells of the initial root explant prior to in vitro regeneration, with these somatic mutations comprising a molecular mutational spectrum differing from that of spontaneous “germline” mutations. This second explanation may be supported by the observation that somatic mutation rates are characteristically higher than germline rates in multicellular organisms [26] and has important particular potential consequences for the evolution of plants, given that they frequently adopt life cycle strategies that involve regeneration from somatic tissues.

Finally, we have highlighted increased frequencies of both base substitutions and small indels in regenerant plants. Regenerant indels mostly occur in homopolymeric or polydinucleotide regions and are probably DNA replication slippage mutations. Intriguingly, combined increases in frequency of both base substitution and replication slippage mutation is characteristic both of the bacterial SOS response [27] and of a subset of human cancer cell lineages [28] and is thought to be due to decreased fidelity of DNA mismatch repair. We suggest that decreased DNA repair fidelity is a major cause of the genetic variability underlying somaclonal variation.

## Experimental Procedures

### *Arabidopsis* Regeneration and Detection of Phenotypic Variance in Regenerant Lineages

Regeneration of R0 plants from root explants from a single (P1) Columbia (Col-0) laboratory strain plant was as described [8]. 28 R0 plants were self-pollinated to generate R1 families (Figure 1A). From each R1 family, 25–30 R1 plants were phenotypically characterized. Phenotypic stability was further monitored in R2 (self-pollination) descendents.

### DNA Sequencing and Sequence Alignment

Genomic DNA from single individuals was sequenced (Illumina Genome Analyzer II technology) using libraries created from ∼350 bp fragments and a single 76 bp paired-end run lane for each sample. Reads were aligned to the TAIR9 reference genome sequence (http://www.arabidopsis.org) using Burrows-Wheeler Aligner [29] (BWA; http://bio-bwa.sourceforge.net/) (Figure S2C).

### Detection of Base Substitutions, Short Insertions, and Deletions

After filtering out all nonuniquely mapped reads and reads with a Phred-scaled mapping quality of less than 20, individual lists of SBSs, short insertions, and short deletions in P1 and the five R1 samples (versus TAIR9) were generated using SAMtools v0.1.5c [30]. Many SBSs and indels were detected in P1 versus TAIR9 (see Figure S2C). As previously reported [31], most of these apparent P1 variants probably represent errors in TAIR9. We detected novel regenerant mutations (mutations in R1 samples and not in P1) using methods summarized in Figure S2C.

### Detection of Large Indels, Chromosomal Inversions, and Translocations

We developed novel codes to detect larger-scale variants (see also [32–35]). Such variants (insertions, inversions, and translocations) will generate “distant-pair” read pairs (where two paired reads [“mates”] align with unexpectedly distant regions of TAIR9 [Figures S2E–S2H]). We therefore extracted reads for which mates mapped >750 bp distant with respect to TAIR9, thus creating new (distant-pair).bam files. Lists of covered regions (depth of coverage of at least five reads) were generated. Visual analysis (using Integrated Genome Viewer (IGV); http://www.broadinstitute.org/igv) [36] of candidates located in noncentromeric regions confirmed eight novel insertions (no inversions or translocations were detected). However, these insertions were present in all P1 and R1 samples (versus TAIR9) and thus not a consequence of regeneration (data not shown).

In addition, systematic comprehensive visual scanning of entire genomes with IGV (window size ∼10 kb) identified mutations including base substitutions, indels (of different sizes), inversions, and translocations (examples in Figures S2I–S2K). These exhaustive searches did not reveal any genomic variants (large or small) in R1 lines additional to the 152 mutations initially identified by the above described IGV-based verification of putative variants in filtered sequence variant lists.

### Validation of Detected Mutations

Standard capillary (“Sanger”) DNA sequencing was used to evaluate false positive mutation detection rates with respect to all detected indels (21 cases in the five sequenced R1 samples) and all base substitutions detected in R1-10-2 and R1-17-2 (74 cases). 20 of the 21 indels (PCR amplification failed in the remaining case) and 70 of the 74 substitutions (PCR amplification failed in the remaining cases) were thus validated (Table S1), indicating negligible false positive detection rates. We also checked our observations with an alternative mutation detection methodology (SHORE [37]). SHORE identified 67 SBSs in R1-10-2, 65 of which are identical to those identified using the methods described in this study (Figures S2O and S2P) and suggesting a negligible overall false negative detection rate in our observations.

### Mutation Rate Estimations

“Regenerant” mutation frequency was estimated as follows. If *n* is the number of identified homozygous mutations (in an R1 plant), 4*n* is the expected number of heterozygous mutations (in the source R0 plant). The mutation frequency *m* per site for an R0 plant is then 4*n*/*s*, where *s* is the (unique reads covered) genome size. Taking R1-1-2 as an example, with n = 20 and *s* = 115.62 Mb, the resulting mutation rate *m* = 4 × 20 / (115.62 × 10^6^) = 6.9 × 10^−7^ is calculated.

### Detection of Transposon Movement

Two strategies were used to search for transposed transposons in regenerant plants. First, variation in read coverage detected transposition-dependent amplification of transposon sequence (see Figure S4). For results shown in Figure 4, the average coverage for each of 3,321 transposable element genes (Table S3) was calculated by summing the coverage at each position within a gene and dividing by the length of the gene, followed by a normalization based on average depth of coverage. We then used IGV (with “distant-pair” analysis; Figure S2E) to check possible amplifications of all transposon sequences for which the Log_2_ coverage ratio score was greater than 0.75. However, the only verified transpositions were those of *AtCOPIA93* previously described in *met1/+ nrpd2* (Figure 4) [20]. No novel transposon amplifications were detected in any of the five sequenced regenerant R1 lines.

Second, any novel transposon insertions (in R1 plants versus TAIR9) would have been expected to cause associated distant-pair reads (as described above), with one read mapping to a region flanking the insertion target site, and the other mapping to a region of the TAIR9 reference containing the original transposon location. Because no such novel distant-pair reads were detected in R1 (versus P1) samples (as described above), we conclude that our regenerant lineages did not detectably carry any novel transposon insertions.

## Supplemental Information

Document S1. Four Figures and One Table

Table S2. List of All Mutations Detected versus P1 Sequence in Five Regenerant Plant Lineages and Genomic Environment of Indel Mutations Related to Figure 2.

Table S3. List of Transposable Element Genes Included in This Study Related to Figure 4.

## References

[1] Birnbaum K.D., Sánchez Alvarado A. (2008). Slicing across kingdoms: Regeneration in plants and animals. Cell.

[2] Humpherys D., Eggan K., Akutsu H., Hochedlinger K., Rideout W.M., Biniszkiewicz D., Yanagimachi R., Jaenisch R. (2001). Epigenetic instability in ES cells and cloned mice. Science.

[3] Skoog F., Miller C.O. (1957). Chemical regulation of growth and organ formation in plant tissues cultured *in vitro*. Symp. Soc. Exp. Biol..

[4] Heinz D.J., Mee G.W.P. (1969). Plant differentiation from callus tissue of *Saccharum* species. Crop Sci..

[5] Larkin P.J., Scowcroft W.R. (1981). Somaclonal variation—A novel source of variability from cell cultures for crop improvement. Theor. Appl. Genet..

[6] Evans D.A., Sharp W.R. (1986). Applications of somaclonal variation. Nat. Biotechnol..

[7] Kaeppler S.M., Kaeppler H.F., Rhee Y. (2000). Epigenetic aspects of somaclonal variation in plants. Plant Mol. Biol..

[8] Valvekens D., Montagu M.V., Van Lijsebettens M. (1988). *Agrobacterium tumefaciens*-mediated transformation of *Arabidopsis thaliana* root explants by using kanamycin selection. Proc. Natl. Acad. Sci. USA.

[9] Atta R., Laurens L., Boucheron-Dubuisson E., Guivarc'h A., Carnero E., Giraudat-Pautot V., Rech P., Chriqui D. (2009). Pluripotency of *Arabidopsis* xylem pericycle underlies shoot regeneration from root and hypocotyl explants grown in vitro. Plant J..

[10] Sugimoto K., Jiao Y., Meyerowitz E.M. (2010). *Arabidopsis* regeneration from multiple tissues occurs via a root development pathway. Dev. Cell.

[11] Gordon S.P., Heisler M.G., Reddy G.V., Ohno C., Das P., Meyerowitz E.M. (2007). Pattern formation during *de novo* assembly of the *Arabidopsis* shoot meristem. Development.

[12] McCoy T.J., Phillips R.L., Rines H.W. (1982). Cytogenetic analysis of plants regenerated from oat (*Avena sativa*) tissue cultures; high frequency of partial chromosome loss. Can. J. Genet. Cytol..

[13] Ossowski S., Schneeberger K., Lucas-Lledó J.I., Warthmann N., Clark R.M., Shaw R.G., Weigel D., Lynch M. (2010). The rate and molecular spectrum of spontaneous mutations in *Arabidopsis thaliana*. Science.

[14] Marriage T.N., Hudman S., Mort M.E., Orive M.E., Shaw R.G., Kelly J.K. (2009). Direct estimation of the mutation rate at dinucleotide microsatellite loci in *Arabidopsis thaliana* (Brassicaceae). Heredity.

[15] Dieterle M., Thomann A., Renou J.P., Parmentier Y., Cognat V., Lemonnier G., Müller R., Shen W.H., Kretsch T., Genschik P. (2005). Molecular and functional characterization of *Arabidopsis* Cullin 3A. Plant J..

[16] Parks B.M., Quail P.H. (1991). Phytochrome-deficient *hy1* and *hy2* long hypocotyl mutants of *Arabidopsis* are defective in phytochrome chromophore biosynthesis. Plant Cell.

[17] Nelson D.C., Lasswell J., Rogg L.E., Cohen M.A., Bartel B. (2000). *FKF1*, a clock-controlled gene that regulates the transition to flowering in *Arabidopsis*. Cell.

[18] Dennis E.S., Brettell R.I.S., Peacock W.J. (1987). A tissue culture induced *Adh1* null mutant of maize results from a single base change. Mol. Gen. Genet..

[19] Slotkin R.K., Martienssen R. (2007). Transposable elements and the epigenetic regulation of the genome. Nat. Rev. Genet..

[20] Mirouze M., Reinders J., Bucher E., Nishimura T., Schneeberger K., Ossowski S., Cao J., Weigel D., Paszkowski J., Mathieu O. (2009). Selective epigenetic control of retrotransposition in *Arabidopsis*. Nature.

[21] Hirochika H., Sugimoto K., Otsuki Y., Tsugawa H., Kanda M. (1996). Retrotransposons of rice involved in mutations induced by tissue culture. Proc. Natl. Acad. Sci. USA.

[22] Peschke V.M., Phillips R.L., Gengenbach B.G. (1987). Discovery of transposable element activity among progeny of tissue culture-derived maize plants. Science.

[23] Murphey P., Yamazaki Y., McMahan C.A., Walter C.A., Yanagimachi R., McCarrey J.R. (2009). Epigenetic regulation of genetic integrity is reprogrammed during cloning. Proc. Natl. Acad. Sci. USA.

[24] Rideout W.M., Eggan K., Jaenisch R. (2001). Nuclear cloning and epigenetic reprogramming of the genome. Science.

[25] Lee M., Phillips R.L. (1987). Genetic variants in progeny of regenerated maize plants. Genome.

[26] Lynch M. (2010). Evolution of the mutation rate. Trends Genet..

[27] McKenzie G.J., Lee P.L., Lombardo M.J., Hastings P.J., Rosenberg S.M. (2001). SOS mutator DNA polymerase IV functions in adaptive mutation and not adaptive amplification. Mol. Cell.

[28] Greenman C., Stephens P., Smith R., Dalgliesh G.L., Hunter C., Bignell G., Davies H., Teague J., Butler A., Stevens C. (2007). Patterns of somatic mutation in human cancer genomes. Nature.

[29] Li H., Durbin R. (2009). Fast and accurate short read alignment with Burrows-Wheeler transform. Bioinformatics.

[30] Li H., Handsaker B., Wysoker A., Fennell T., Ruan J., Homer N., Marth G., Abecasis G., Durbin R., 1000 Genome Project Data Processing Subgroup (2009). The Sequence Alignment/Map format and SAMtools. Bioinformatics.

[31] Ossowski S., Schneeberger K., Clark R.M., Lanz C., Warthmann N., Weigel D. (2008). Sequencing of natural strains of Arabidopsis thaliana with short reads. Genome Res..

[32] Korbel J.O., Urban A.E., Affourtit J.P., Godwin B., Grubert F., Simons J.F., Kim P.M., Palejev D., Carriero N.J., Du L. (2007). Paired-end mapping reveals extensive structural variation in the human genome. Science.

[33] Sudmant P.H., Kitzman J.O., Antonacci F., Alkan C., Malig M., Tsalenko A., Sampas N., Bruhn L., Shendure J., Eichler E.E., 1000 Genomes Project (2010). Diversity of human copy number variation and multicopy genes. Science.

[34] Medvedev P., Stanciu M., Brudno M. (2009). Computational methods for discovering structural variation with next-generation sequencing. Nat. Methods.

[35] Alkan C., Sajjadian S., Eichler E.E. (2011). Limitations of next-generation genome sequence assembly. Nat. Methods.

[36] Robinson J.T., Thorvaldsdóttir H., Winckler W., Guttman M., Lander E.S., Getz G., Mesirov J.P. (2011). Integrative genomics viewer. Nat. Biotechnol..

[37] Schneeberger K., Ossowski S., Lanz C., Juul T., Petersen A.H., Nielsen K.L., Jørgensen J.E., Weigel D., Andersen S.U. (2009). SHOREmap: Simultaneous mapping and mutation identification by deep sequencing. Nat. Methods.

