# Supplementary material for: Regenerant Arabidopsis Lineages Display a Distinct Genome-Wide Spectrum of Mutations Conferring Variant Phenotypes
Source: Curr Biol. 2011 Aug 23;21(16):1385–90. doi: 10.1016/j.cub.2011.07.002 (PMC3162137; doi:10.1016/j.cub.2011.07.002)
Supplement: Document S1. Four Figures and One Table [file mmc1.pdf]

Current Biology, Volume *21*

Supplemental Information

Regenerant *Arabidopsis* Lineages Display a Distinct Genome-Wide Spectrum of Mutations Conferring Variant Phenotypes

Caifu Jiang, Aziz Mithani, Xiangchao Gan, Eric Belfield, John P. Klingler, Jian-Kang Zhu, Jiannis Ragoussis, Richard Mott, and Nicholas P. Harberd

Inventory of Supplemental Information

Figure S1. A Heritable Unstable Variant Regeneration-Induced Phenotype. Related to Figure 1.
Figure S2. Genome-Wide Analysis of Mutations in Regenerant Plants. Related to Figure 2. Provides detailed information concerning analysis of next-generation short read sequencing data, mutation calling, etc.
Figure S3. Complementation Test Allelism Verifications of Novel Mutant *HY1* and *FKF1* Alleles. Related to Figure 3.
Figure S4. Depth of Read Coverage in the Vicinity of *AtCOPIA 93* (AT5G17125). Related to Figure 4. Shows that the translocation of *AtCOPIA 93* in *met1*/+ *nrpd2* resulted in an increase in the depth of *AtCOPIA 93* read coverage.

Table S1. Phenotypic Description of Regenerant Lines. Related to Figure 1. Provides phenotypic information concerning each regenerant line.

Table S2. List of All Mutations Detected (versus P1 sequence) in Five Regenerant Plant Lineages and Genomic Environment of Indel Mutations. Related to Figure 2. Provides detailed information concerning each of the mutations detected in the five regenerant plants (Table S2A). Also shows differences in the genomic environments of regenerant indels versus indels arising spontaneously in sexually propagated plants (Table S2B).
Table S3. List of Transposable Element Genes Included in This Study. Related to Figure 4. (excel file).

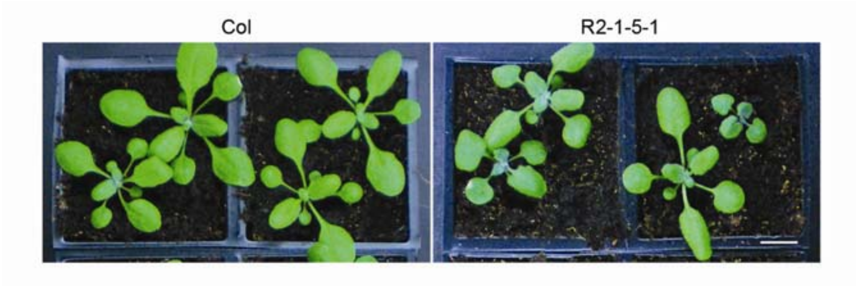

Figure S1. A Heritable Unstable Variant Regeneration-Induced Phenotype. Related to Figure 1.

Left: Col control. Right: Self-pollination of plant R1-5-1, a plant exhibiting a compact leaf phenotype, generated the R2 family shown. The phenotype was heritable but highly variable in intensity and with a segregation ratio divergent from Mendelian expectation. Siblings of R1-5-1 exhibited the same phenotype and transmitted it to subsequent generations in a similar non-Mendelian fashion. These observations are all suggestive of allelic instability. Plants are 35 days old. Bar = 1cm.

A

Lines	Reads (millions) Raw	Reads (millions) Aligned	Extent of genome covered (Mb)	Estimated coverage
P1	5.62	4.13	115.77	27.11
R1-1-2	4.94	3.79	115.62	24.91
R1-6-2	5.20	3.96	115.64	26.03
R1-10-2	5.93	4.52	115.65	29.70
R1-17-2	5.12	3.89	115.62	25.57
R1-19-2	4.58	3.49	115.59	22.95

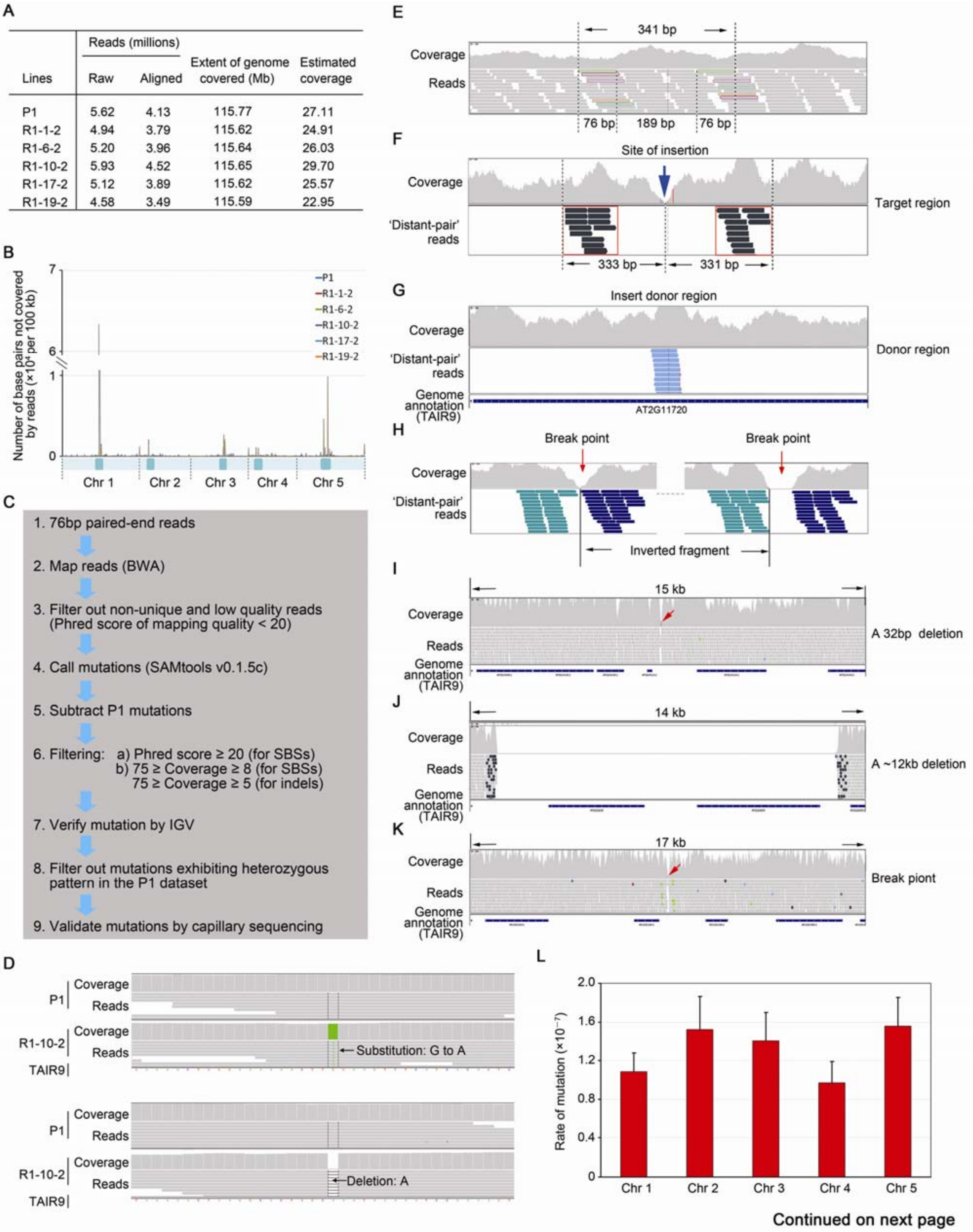

Continued on next page

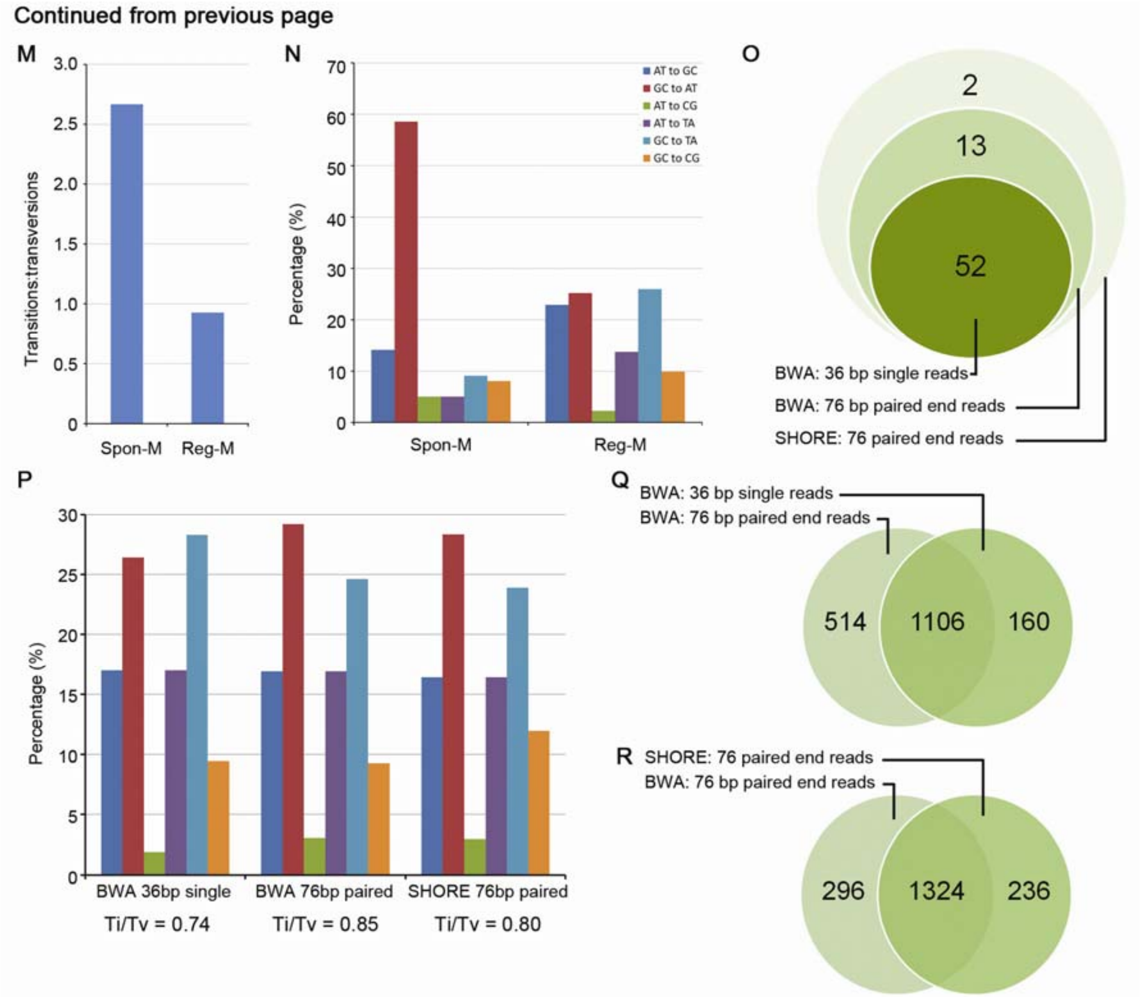

Figure S2. Genome-Wide Analysis of Mutations in Regenerant Plants. Related to Figure 2.

(A) Total number of sequencing reads, aligned reads, and extent of genome coverage in genomic DNA sequence datasets from P1 and R1 lines.

(B) Distribution of the number of base pairs not covered by uniquely mapped reads in P1 and R1 datasets. Centromere regions are represented in darker blue.

(C) Schematic representation of methods for detection of novel mutations in R1 (versus P1) datasets. (1) 76bp paired-end reads were generated by Illumina Genome Analyzer II technology. (2) Reads were mapped to TAIR9 using BWA [29]. (3) Non-unique and low quality reads were filtered out. (4) Mutations were called using SAMtools v0.1.5c [30]. (5) All P1 variants (1572 SBSs and 1620 indels versus TAIR9) were subtracted from R1 lists. (6) Mutations with a Phred score <20 or that failed coverage filtering (criteria for SBSs: 75 ≥ coverage ≥8; criteria for indels: 75 ≥ coverage ≥ 5) were rejected. (7) Mutations were accepted (homozygous mutations) only when ≥95% of reads were seen using IGV (http://www.broadinstitute.org/igv) to contain the same sequence difference with respect to P1 sequence (examples as shown in D). (8) All accepted mutations that exhibited a heterozygous pattern in P1 were also excluded from the final mutation lists. (9) Subsets of detected mutations were validated by capillary sequencing. These methods enabled detection of regenerant

homozygous base substitutions and short indels within R1 plants by excluding all variants (versus TAIR9) inherited from the P1 progenitor.
(D) Examples showing IGV-based verification of base substitution and indel variants.
(E-G) The effects of insertion on read coverage and read-pair relationships as visualized in IGV. P1 reads are aligned against TAIR9 reference sequence.
(E) Read-pair relationships (76bp paired-end data). Reads represented in the same color are part of the same pair. As ~350bp fragments were used to construct the 'paired-end' library, each pair of 'paired-end' reads typically represent 76bp fragments, one from the left (forward read) and one from the right (reverse read) end of the corresponding ~350bp genomic DNA fragment.
(F) An insertion target site. An insertion (in P1 versus TAIR9) causes a local reduction in depth of coverage (highlighted by the arrow). In addition, groups of 'distant-pair' reads (reads for which pairs align with TAIR9 at >750bp distance from one another) flank the insertion site on either side. Reads shown in the red boxes are example 'distant-pair' reads that singly align with the illustrated region of the reference genome but whose mate pairs do not align in the vicinity (within 750bp).
(G) Donor region (genomic region of origin of the sequence inserted in (F)). Blue reads are 'distant-pair' reads: these reads pair with the reads shown in the red boxes in F.
(H) The effects of inversion on read coverage and read-pair relationships as visualized in IGV. Two chromosomal break points (highlighted by the red arrows) caused by a novel simple interstitial chromosomal inversion are shown. In the region of each break point there is a local reduction in the depth of coverage. Flanking each break point are groups of 'distant-pair' reads. 'Distant-pair' reads and associated mate pairs are shown in the same color for each group.
(I-K) Mutation detection via IGV-based systematic comprehensive untargeted visual scanning of entire genomes. Red-bordered reads are reads whose mates were not mapped. Fill-coloured reads are 'distant-pair' reads. Red arrows highlight the location of mutations.
(I) A 32bp deletion. Note the local reduction in depth of coverage at the location of the deletion.
(J) A ~12kb deletion in a 14kb window.
(K) A break point (the result of insertion, inversion or translocation; highlighted by the red arrow). Note the local reduction in depth of coverage at the location of the break point.
(L) Chromosome-specific mutation rate across R1 lines. Error bars are standard errors of means. No significant between chromosome mutation rate differences were detected (G test, p-value = 0.40).
(E-G) and (K) align reads from P1 plants, (H-J) align reads from a separate experiment involving material derived from the Landsberg *erecta* laboratory strain (data not shown) and thus represent genetic differences between Landsberg *erecta* and the Col-0 reference.
(M-R). Relative frequencies of spontaneous versus regenerant base substitution and indel mutation classes. (M) Transition: transversion ratios for mutations occurring spontaneously in sexually propagated plants (Spon-M; [13]) and in regenerant plants (Reg-M; data from this paper).
(N) Percentage of mutations in each of the six possible mutation classes (data as in M).
(O-R) Evaluation of the effects of different kinds of DNA sequence datasets (36bp single reads (as used in [13]), versus 76bp paired-end reads (as used in this study)) and the alternative data analysis methods described in this study versus SHORE [37]

(as used in [13]) on SBS and Indel detection. These evaluations confirm the legitimacy of comparing mutation rates observed in this study with those in [13] and of the comparisons shown in Figures 2D and 2E and in (M) and (N) in this figure.

(O) Dataset comparisons: SBS detection. The reads from the 76bp paired-end read R1-10-2 dataset were computationally reduced in length, thus generating a derived 36bp single read dataset. Analysis of this converted dataset using the SBS detection methods described in this study identified 52 SBSs (versus P1), meaning that 13 of the 65 SBSs originally detected in the initial 76bp paired-end read dataset (Figure 2B) were now not detected. These 13 detection failures occurred because the 36bp single read dataset contained no reads uniquely mapping to the relevant locations of TAIR9, causing zero coverage of the regions carrying the undetected SBSs. Thus use of larger read lengths and utilization of pair-end reads increases the power of SBS detection. However, the extent of this increase is insufficient to contribute more than marginally to the orders of magnitude difference in mutation rate reported in this study versus that in [13]. Further analysis of the R1-10-2 76bp paired-end read dataset using SHORE [37] to detect SBSs (versus P1) identified 67 SBSs (including the 65 previously identified). Thus SHORE identified 2 SBSs additional to those previously detected by the BWA-based methods used in this study. One of these additional mutations was detected by SHORE but not by our methods because only 7 reads carried this mutation (at least 8 reads carrying the mutation are needed to call a SBS in our methods). We missed the other additional SBS because it was adjacent to a homopolymeric stretch. However, these small differences in bioinformatic variant detection are also unlikely to contribute substantially to the differences between the mutation rates reported in this study and in reference [13].

(P) Molecular mutation class distribution of mutations detected with varying datasets and data analysis methods used in (O). In all three cases shown a very similar distribution (spectrum) is detected in R1-10-2. Hence differences in dataset properties or methods analysis contribute negligibly to the differences between the molecular mutation spectrum reported in this study (Figure 2D) and that reported in [13].

(Q) Dataset comparisons: Indel detection. The reads from the 76bp paired-end read P1 dataset were computationally reduced in length, thus generating a derived 36bp single read dataset. Indel detection using the methods described in C (steps1-6) identified 1266 indels (versus TAIR9) in this derived dataset, compared with the 1620 identified in the 76bp paired end data. In all, 1106 indels were identified in both datasets, 160 were identified in the derived dataset only, and 514 were identified in the 76bp paired end data only. These results indicate that use of larger read lengths and utilization of pair-end reads increases the power of indel detection. However, the extent of this increase is insufficient to contribute more than marginally to the increased frequency of indel mutations reported in this study versus those in [13].

(R) Methods comparisons: Indel detection. Analysis of the P1 76bp paired-end read dataset using SHORE [37] to detect indels (versus TAIR9) identified 1560 indels (of which 1324 overlapped with those detected using the methods described in C (steps1-6)). These observations indicate that the two types of data analysis have differing strengths and weaknesses with respect to indel detection. However, these differences are also insufficient to contribute substantially to the differences between the mutation rates reported in this study and in reference [13]. In a further investigation of indel frequencies, we have sequenced plants representative of six Col-0 mutation accumulation lines (grown in a greenhouse for five generations) using 100bp paired-end sequencing technology, and analyzed the data using the methods described in C. We detected two novel indels (an A and an AT insertion; data not

shown). This detected indel mutation rate is comparable with that in [13], suggesting that the studies reported therein had not significantly failed to detect spontaneous indel mutations that would have been detectable using our methods, hence further indicating that comparison of indel mutation rates in our study with those in reference [13] is reasonable.

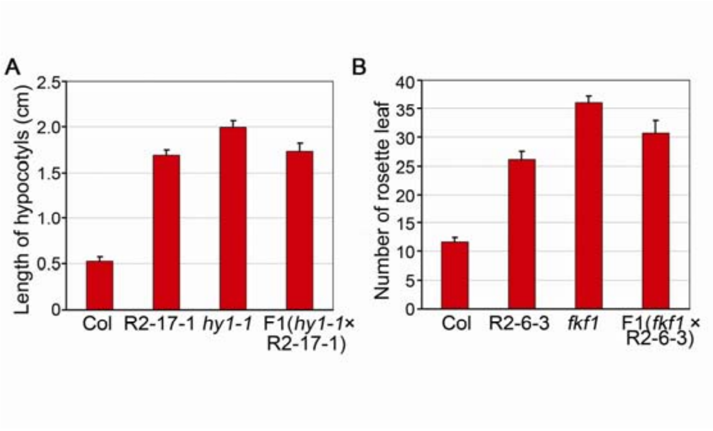

Figure S3 Complementation Test Allelism Verifications of Novel Mutant *HY1* and *FKF1* Alleles. Related to Figure 3. (A) Hypocotyl lengths, genotypes as indicated. (B) Flowering times (expressed as number of leaves in the vegetative rosette), genotypes as indicated. Error bars represent standard errors of means.

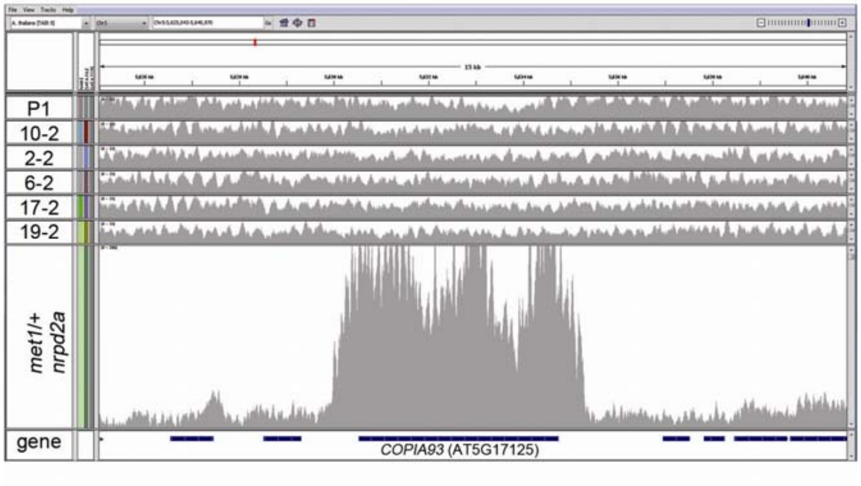

Figure S4. Depth of Read Coverage in the Vicinity of *AtCOPIA 93* (AT5G17125). Related to Figure 4. Illumina Genome Analyzer reads from P1 progenitor, the 5 different R1 lines (10-2, 2-2, etc.) and *met1*/+ *nrpd2* [20] samples were aligned with the TAIR9 reference *A. thaliana* (Col-0) sequence and visualised using Integrated Genome Viewer (IGV) software. The y-axis measures depth of coverage, which is elevated for *AtCOPIA93* in *met1*/+ *nrpd2*, but not in the other samples.

Table S1. Phenotypic Description of Regenerant Lines. Related to Figure 1.

Lines	Phenotype of R1 plants	Heritability of phenotype
1	No visible phenotype	
2	No visible phenotype	
3	No visible phenotype	
4	No visible phenotype	
5	Compact leaf, no segregation	Phenotype heritable, but not stable
6	Late flowering, segregating	Phenotype heritable and stable
7	No visible phenotype	
8	Big flower, segregating	Phenotype heritable, but not stable
9	No visible phenotype	
10	Bleached cotyledon, segregating	Lethal
11	No visible phenotype	
12	No visible phenotype	
13	Abnormal leaf, segregating	Phenotype heritable and stable
14	No visible phenotype	
15	No visible phenotype	
16	No visible phenotype	
17	Long hypocotyl, segregating	Phenotype heritable and stable
18	No visible phenotype	
19	Late flowering, segregating	Phenotype heritable and stable
20	No visible phenotype	
21	No visible phenotype	
22	No visible phenotype	
23	No visible phenotype	
24	No visible phenotype	
25	Small, segregating	Phenotype heritable and stable
26	No visible phenotype	
27	No visible phenotype	
28	No visible phenotype	

Table S2 List of All Mutations Detected (versus P1 sequence) in Five Regenerant Plant Lineages and Genomic Environment of Indel Mutations. Related to Figure 2. (excel file)

Table S3. List of Transposable Element Genes Included in This Study. Related to Figure 4. (excel file)
